# Randomized controlled trial of mailed Nicotine Replacement Therapy to Canadian smokers: study protocol

**DOI:** 10.1186/1471-2458-11-741

**Published:** 2011-09-28

**Authors:** John A Cunningham, Scott T Leatherdale, Peter L Selby, Rachel F Tyndale, Laurie Zawertailo, Vladyslav Kushnir

**Affiliations:** 1 Centre for Addiction and Mental Health, 33 Russell St., Toronto, M5S 2S1, Canada; 2 Department of Psychology, University of Toronto, 100 St. George St., Toronto, M5S 3G3, Canada; 3 Dalla Lana School of Public Health, University of Toronto, 155 College St., Toronto, M5T 3M7, Canada; 4 Department of Health Studies and Gerontology, University of Waterloo, 200 University Ave., Waterloo, N2L 3G1, Canada; 5 Department of Population Studies and Surveillance, Cancer Care Ontario, 620 University Ave., Toronto, M5G 2L7, Canada; 6 Department of Pharmaceutical Sciences, University of Toronto, 144 College St., Toronto, M5S 3M2, Canada; 7 Department of Family and Community Medicine, University of Toronto, 500 University Ave., Toronto, M5G 1V7, Canada; 8 Department of Pharmacology and Toxicology, University of Toronto, 1 King's College Circle, Toronto, M5S 1A8, Canada; 9 Department of Psychiatry, University of Toronto, 250 College St., Toronto, M5S 1A8, Canada

## Abstract

**Background:**

Considerable public health efforts are ongoing Canada-wide to reduce the prevalence of smoking in the general population. From 1985 to 2005, smoking rates among adults decreased from 35% to 19%, however, since that time, the prevalence has plateaued at around 18-19%. To continue to reduce the number of smokers at the population level, one option has been to translate interventions that have demonstrated clinical efficacy into population level initiatives. Nicotine Replacement Therapy (NRT) has a considerable clinical research base demonstrating its efficacy and safety and thus public health initiatives in Canada and other countries are distributing NRT widely through the mail. However, one important question remains unanswered - do smoking cessation programs that involve mailed distribution of free NRT work? To answer this question, a randomized controlled trial is required.

**Methods/Design:**

A single blinded, panel survey design with random assignment to an experimental and a control condition will be used in this study. A two-stage recruitment process will be employed, in the context of a general population survey with two follow-ups (8 weeks and 6 months). Random digit dialing of Canadian home telephone numbers will identify households with adult smokers (aged 18+ years) who are willing to take part in a smoking study that involves three interviews, with saliva collection for 3-HC/cotinine ratio measurement at baseline and saliva cotinine verification at 8-week and 6-month follow-ups (N = 3,000). Eligible subjects interested in free NRT will be determined at baseline (N = 1,000) and subsequently randomized into experimental and control conditions to receive versus not receive nicotine patches. The primary hypothesis is that subjects who receive nicotine patches will display significantly higher quit rates (as assessed by 30 day point prevalence of abstinence from tobacco) at 6-month follow-up as compared to subjects who do not receive nicotine patches at baseline.

**Discussion:**

The findings from the proposed trial are timely and highly relevant as mailed distribution of NRT require considerable resources and there are limited public health dollars available to combat this substantial health concern. In addition, findings from this randomized controlled trial will inform the development of models to engage smokers to quit, incorporating proactive recruitment and the offer of evidence based treatment.

**Trial Registration:**

ClinicalTrials.gov: NCT01429129

## Background

Smoking is a leading cause of preventable death worldwide and the leading cause of preventable cancer death. Tobacco use is responsible for approximately 33% of potential years of life lost (PYLL) due to all cancers in males and approximately 20% of PYLL due to all cancers in females [1]. In males, it is also responsible for approximately 30% of PYLL due to diseases of the heart and 50% of PYLL due to respiratory diseases [1]. In 2002, over 2 million acute care hospital days (10.3% of all acute care hospital days) in Canada were attributable to smoking [2]. The cost of these smoking-attributable acute care hospital days exceeded $2.5 billion.

Approximately 18% of the adult Canadian population (20% of males and 16% of females) were identified as current smokers in 2008. Although this proportion has declined from 25% in 1999, it still represents 4.9 million Canadian smokers [3]. Quitting smoking conveys numerous immediate, intermediate and long term health benefits. For example, coronary heart disease risk is reduced by 50% after 12 months without smoking, and after 15 years the risk is as low as that of a non-smoker. Ten years after quitting the mortality rate from lung cancer is about half that of a continuing smoker [4]. Helping smokers quit smoking should be a public health priority.

In a systematic assessment of the value of clinical preventive services recommended by the U.S. Preventive Services Task Force, smoking cessation treatment for adults was one of the highest ranked services in terms of its cost effectiveness and its potential to reduce the burden of disease [5,6]. Smoking cessation services compare favorably with other routine preventive health care interventions such as screening for hypertension and annual mammography. Most smoking cessation interventions cost less per year of life saved than most widely accepted medical practices. For example, cost effectiveness analysis of the implementation of the Agency for Healthcare Research and Quality (AHRQ) [7] guidelines show costs of $4,113 per life-year saved, in 2001 prices. This compares favorably to annual mammography for women aged 40 to 49 years, which costs $71,751 in 2001 prices, and hypertension screening for men aged 40+ years, which costs $27,117 in 2001 prices. A British systematic analysis of smoking cessation interventions found that Nicotine Replacement Therapy (NRT) costs between £1000 and £2400 per year of life saved [8].

Large scale consumer consensus and population research conducted across parts of the U.S. and Canada has shown that mailed free NRT is an effective way of promoting smoking cessation and of helping motivated smokers who want to quit. Research conducted in New York State found that 53% of smokers indicated that the free distribution of NRT would be most effective in motivating them to think seriously about stopping smoking [9]. A Canadian population survey similarly found that 58.9% of ≥ 10 cigarettes per day smokers expressed interest in receiving free nicotine patches and of those, almost all indicated they would use nicotine patches to quit permanently [10]. Heavier smokers were more likely than less heavy smokers to be interested in receiving free NRT.

In an effort to increase access to smoking cessation therapies, one program in New York State provided over 40,000 smokers who called in to a Smokers' Quitline with one to six weeks of free nicotine patches or vouchers for two weeks of NRT. Evaluation of the giveaway program revealed that Quit rates four months later varied from 21% (among smokers receiving one week of NRT for free) to 35% (among smokers receiving 6 weeks of NRT for free). This compared with a quit rate of 12% among an earlier comparison group of callers to the Quitline (not randomly assigned) who received counseling support and some self-help materials but no NRT [11]. In Ontario, Canada, the STOP Study has distributed free NRT and thus far has been responsible for the delivery of 5 weeks of free NRT to approximately 58,000 smokers (representing roughly 33% of adult Ontario smokers who smoke ≥ 10 cigarettes a day and who report wanting to quit in the next 30 days) [12]. As part of a call centre-based mass distribution initiative, end-of-treatment quit rate (using 7 day point prevalence) was estimated to be 15% (the calculation used as the denominator all those who were sent NRT and assumed that all recipients who did not complete their end-of-treatment survey had not quit). At the 6-month follow-up period, the quit rate based on 30 day point prevalence was estimated to be approximately 9% [12]. Of particular note, the majority (76%) of smokers in the STOP Study requested nicotine patches as their desired form of NRT.

Overall, the existing research suggests that smokers are interested in easier access to smoking cessation therapies, and that the receipt of such therapies, including free NRT, seems to be associated with increased rates of quitting smoking. However, none of the studies exploring increased availability of NRT through mass distribution were randomized controlled trials. As a result, the findings are suggestive but do not permit causal inference. Additionally, biochemical verification of self-report abstinence was not performed in these studies. Considerable money has been invested in mass distribution of free NRT in Ontario and similar initiatives are being undertaken or contemplated in other provinces. It is important at this stage to conduct a randomized controlled study to determine the efficacy of large scale distribution of free NRT.

### Aim of the study

This study will attempt to answer the question, "does mass distribution of the nicotine patch actually work (i.e., increase quit rates significantly above those who do not receive free nicotine patches)?" By conducting a randomized controlled trial of free mass NRT distribution, this study will evaluate the efficacy of the mass distribution approach. Comparison of an experimental group which will be offered free nicotine patches to a control group that does not get offered nicotine patches will determine whether or not mass distribution of NRT is an effective way of helping smokers quit. It is hypothesized that among eligible Canadian smokers expressing interest in receiving free nicotine patches, those randomly selected to be offered 5 weeks of free nicotine patches will exhibit a significantly greater quit rate at 8 weeks and 6-month follow-ups compared to those not offered free nicotine patches (saliva cotinine measurements used to validate self-reported smoking abstinence). Previously it has been shown that those who metabolize nicotine more quickly have significantly poorer cessation rates on nicotine patch than slow metabolizers [13]. Therefore this study will also explore whether fast nicotine metabolizers (as measured by the 3-Hydroxycotinine/cotinine ratio) will be less likely to succeed at quitting smoking, as compared to slow nicotine metabolizers, when offered free nicotine patches. Finally, the study will investigate how compliance with recommended amount of NRT, and whether prior use of NRT, affects success at quitting smoking when smokers are offered free nicotine patches.

## Methods/design

### Design

This is a single-blind, randomized controlled trial of mass distribution of free Nicotine Replacement Therapy to Canadian smokers. A two-stage recruitment process will be employed, in the context of a general population survey with two follow-ups (8 weeks and 6 months). Random digit dialing of Canadian home telephone numbers and an initial interview will identify households with adult (age 18 or over) smokers who smoke 10 or more cigarettes a day. One individual from each household who is willing to take part in a smoking study that involves three interviews, with saliva collection for 3-HC/cotinine ratio measurement at baseline and saliva cotinine verification at 8-week and 6-month follow-ups will be randomly selected (according to most recent birthday). Willing participants will be offered a $20 honorarium for completing the baseline and each of the 8-week and 6-month follow-ups. Verbal consent will be obtained as the initial contact is by telephone.

**Figure 1:**
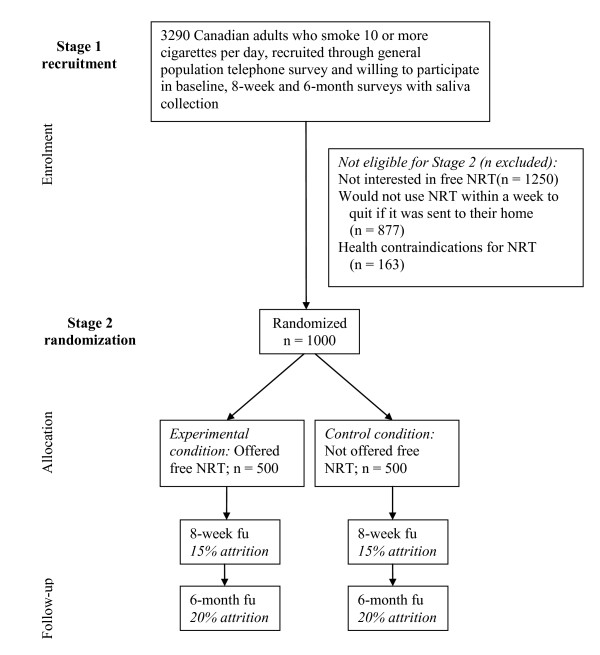
**Overview of the proposed intervention trial**.

As part of the baseline survey, eligible subjects will be identified for the second stage of the recruitment process and will be randomized into experimental and control conditions to receive versus not receive nicotine patches. Eligibility will be determined by a series of questions regarding hypothetical interest in nicotine patches to quit smoking (including willingness to have nicotine patches sent to their home) and having no contraindications for using NRT. A randomized half of the eligible subjects will be assigned to the experimental condition and asked for their permission to have nicotine patches sent to their home. These subjects will have 5 weeks of nicotine patches sent to their homes to help them quit smoking. Subjects in the control condition will not be offered nicotine patches. Only subjects in the randomized controlled trial will be followed-up at 8 weeks and 6 months. See Figure 1 for a CONSORT diagram of the proposed study design.

### Ethical approval

The research methods to be used in this study have been approved by the standing ethics review committee of the Centre for Addiction and Mental Health.

### Participants - inclusion and exclusion criteria

Participants will be recruited using a general population telephone survey of Canadian residents. A two stage recruitment procedure will be employed.

#### Criteria for Participation in baseline survey

Participation in the baseline recruitment survey will be restricted to current smokers, age 18 and above, who smoke 10 or more cigarettes per day, who are interested in being involved in a smoking study, and who are willing to answer three sets of questions: now, after 8 weeks and after 6 months. In addition, participants must be willing to provide a saliva sample for cotinine analysis at each time point (sent by mail [14,15]; note that 97% of cotinine samples collected by mail were usable in these studies).

#### Criteria for Assignment to the Randomized Controlled Trial

As part of the baseline survey, all subjects will be asked a series of questions to assess their level of interest in receiving free NRT: "The Ministry of Health is considering different ways to help people stop smoking. One option would be to provide interested smokers with free Nicotine Patches. If Nicotine Patches were offered for free, would you be interested in receiving them?" Those who say "yes" will then be asked if they would use the nicotine patch to quit smoking. Those who say "yes" to this question will be asked if they would begin to use the patch within 1 week of receiving it. A "yes" to this question will lead to being asked if they would be willing to have the Patch sent to their home. These questions have been used in a previous survey and form the basis of the sample size estimate for the present study [10]. Finally, as part of the demographic section of the survey, subjects will also be asked questions regarding health conditions contraindicating NRT use (i.e., being pregnant, intending to become pregnant, or breastfeeding; having a serious heart or circulation problem, not including high blood pressure). Subjects will be eligible for the randomized controlled trial if they are interested in NRT, would use it to quit within 1 week of receipt, are willing to have the NRT sent to their home, and have no health contraindications for the use of NRT. Subjects who meet all these eligibility criteria will then be randomly assigned to the experimental or control condition.

Although some participants will report that they have used NRT in the past, they will not be excluded from the study due to prior NRT use because the intent is to evaluate the impact of NRT in the full range of potential community participants. In addition, our previous research has indicated that prior use of NRT is actually positively related to current interest in using NRT again [10]. Thus, one of the secondary analyses of this study will test the moderating hypothesis that prior use of NRT is negatively related to future success with NRT when it is offered for free.

### Interventions

#### Experimental condition

A randomized half of the subjects meeting baseline criteria and expressing an interest in receiving free nicotine patches, willingness to use the Patches within a week to quit smoking, interest in having the patches sent to their home, and meeting criteria for eligibility to receive NRT (i.e., no health contraindications) will be randomly assigned to the experimental condition (the remainder will be assigned to the control condition). Subjects in the experimental condition will be asked, at the end of the baseline survey, if they want to be sent a free, 5-week supply of Nicotine Patches. Those who say "yes" will have the NRT mailed to their home the week after their baseline interview. As is employed in the Ontario mass distribution of NRT initiative [12], a 5-week program of nicotine patches will be sent (3 weeks of Step 1 [21 mg of nicotine]; 1 week of Step 2 [14 mg of nicotine]; 1 week of Step 3 [7 mg of nicotine]). Once randomly assigned to this condition, subjects will be counted as part of the experimental condition whether they agree to accept the NRT or not (intent-to-treat approach).

The 5-week program of nicotine patches was chosen for pragmatic reasons, which balanced a limited budget to supply the NRT and conduct a large population survey, and because of findings from literature regarding effective treatment duration. A recent Cochrane review evaluating the efficacy of NRT has revealed no differences in treatment effect between trials offering up to eight weeks of nicotine patch treatment and those offering longer treatments [16]. Furthermore, several trials evaluating usage patterns of free nicotine patches offered to smokers through a Smokers' Quitline have shown no differences in 7- and 12-month quit rates between individuals receiving 2, 4, 6, or 8-week supplies of free nicotine patches [17,18]. These findings however, are inconsistent with two other studies demonstrating higher quit rates among Smokers' Quitline callers receiving 8 weeks versus 2 weeks of free nicotine patches [19], and 6 weeks versus 4 weeks of free NRT [20]. Given the apparent inconsistencies in the literature surrounding effective treatment duration of mailed out free NRT, we chose a middle of the road approach of 5 week nicotine patch program.

#### Control condition

*S*ubjects randomly assigned to the control condition will not be asked, at the end of the baseline survey, if they want free nicotine patches sent to their homes. An advantage of the design procedure to be employed in this trial is that respondents in the control condition will not have the expectation that they will receive NRT. Thus, their smoking outcomes at 8-week and 6-month follow-ups will reflect a true natural history comparison to the outcomes of subjects in the experimental condition.

An alternate control condition would be to provide subjects with placebo patches. However, the objective of this trial is to test the question, "does mailed distribution of free NRT (in the form of the nicotine patch) lead to increased quit rates among Canadian smokers?" This objective is a pragmatic one and requires the random assignment of offering free NRT to some and nothing to others. Whether the impact of the NRT is due to the active ingredient or due to a placebo effect is irrelevant to this objective. Thus, the most appropriate control condition is one where subjects receive nothing, and further, have no expectation that they would receive anything.

#### Content of Baseline Interview

The primary purpose of the baseline interview is to assess eligibility for the randomized controlled trial. Specifically, subjects will be asked of their interest in receiving free NRT, its use within one week of receipt, and health contraindications for NRT use. In addition, other questions will also investigate the following: a) subjects' heaviness of smoking [21], b) number and duration of past quit attempts, c) past use of NRT, d) current life stressors, e) motivation to quit smoking, f) past and present drug use, g) level of alcohol intake (AUDIT-C) [22], h) presence of mental health disorders diagnosis, and e) a series of demographic characteristics such as age, gender, ethnicity, marital status, education and employment status.

#### Content of 8-week and 6-month follow-up interviews

At 8-week and 6-month follow-ups, subjects who have not quit will be asked how many cigarettes per day they currently smoke and their intentions to quit smoking; how soon after waking they smoke their first cigarette; and if, since their baseline interview, they have stopped smoking for even one day because they were trying to quit. Subjects in the experimental condition will be asked to evaluate their experiences with NRT and to elaborate on their efforts to quit smoking. Specifically, at 8-weeks subjects will be asked if they received the NRT; how soon after receipt they began to use it; how much of it (if any) they used; if relevant, why they did not use any or all of it; whether or not they experienced any withdrawal symptoms and which ones; and what challenges they encountered to quitting/reducing. At 6-months, subjects will be asked whether or not they purchased additional NRT to help in their quit effort (what type and how much); whether or not they plan to purchase more NRT to quit or maintain their non-smoking; if they have smoked in the past 30 days or reduced from baseline, what benefits they have noticed; what supports they had for quitting; and what additional resources they sought to help them quit. These questions will be asked after the core smoking status outcome measures have been assessed. Subjects in the control condition will also be asked about their use of NRT but the questions will be framed to reflect purchase of NRT from sources other than this ongoing study. Subjects in both conditions will also be asked about their use of other smoking cessation aids.

#### Validation of self-report by saliva cotinine samples

Several studies have demonstrated the utility of collecting saliva cotinine samples by mail using the same Salivette sampling method proposed for this study [14,15]. While clinically tobacco exposure is measured by quantifying cotinine in either plasma or urine, salivary cotinine is a reliable measure of nicotine or tobacco exposure that is widely used in both clinical and epidemiological or population-based studies [23-25]. Participants will be asked to collect their saliva sample at baseline and on the day they complete their follow-up interviews (saliva collection kits mailed several days following baseline interview and one week before each follow-up interview is due, along with a reminder letter about the upcoming telephone interview). At baseline, the saliva sample will allow for the calculation of the 3-HC/cotinine ratio to test the moderating hypothesis of nicotine metabolism rate on success at tobacco cessation using nicotine patches. Collection of saliva samples at follow-up periods will allow for the verification of smoking abstinence.

### Randomization

Recruitment is conducted using a Random Digit Dialing survey with computer assisted telephone interview (CATI) technology. At the end of the baseline interview, eligible subjects will be allocated to experimental and control conditions using a simple randomization without replacement built into the CATI program (no stratification or minimization given the large proposed sample size in this trial).

### Blinding

Interviewers will be blind to subjects' condition because they will be using the CATI technology. As the first parts of the 8-week and 6-month follow-ups are identical for experimental and control conditions, they will not know the intervention condition to which a subject belongs until questions specific to the use of NRT are asked near the end of the surveys (i.e., after the primary outcome measures are assessed). Further, while it is possible that a participant might 'unblind' themselves to an interviewer by volunteering that they used the nicotine patch, this is still unlikely to have an impact as the interviewers will be part of a separately contracted telephone survey research firm and will not be aware of the hypotheses of this research trial.

### Outcome Measures

#### Primary outcome measures

The primary outcome measure will be 30 day point prevalence of abstinence at 6 months, validated by saliva cotinine samples.

#### Secondary outcome measures

Secondary outcome measures are: (1) 7 day point prevalence of smoking abstinence at 8 weeks post-baseline; (2) reduction in smoking since baseline; (3) compliance with the full 5-week course of nicotine patches provided to experimental condition; (4) challenges with quitting/reducing smoking; and (5) purchase and use of NRT and smoking cessation aids at follow-up periods.

### Data Analysis

#### Power analysis

Quit rates for those who use nicotine patches have been obtained from several sources, including systematic reviews by Fiore et al. [26] and a Cochrane review by Stead and colleagues [16]. Both these reviews indicate a 10% increase in tobacco cessation rates upon using nicotine patches as compared to placebo at 6-month follow-up. This 10% increase is roughly in concordance with the 30 day point prevalence rate for abstinence observed at the 6-month follow-up point of the STOP mass distribution demonstration trial (9%; note that this trial had no control group so the results reflect pre-post changes in smoking rates) [12]. However, we recognize that both of these estimates are probably too large for a pragmatic randomized controlled trial because the impact of interventions is often smaller in real world settings (and pre-post estimates, such as those found in the STOP Study, can also be inflated). Thus, we have chosen to power our trial based on the assumption that our quit rate will be half as large (i.e., a 5% increase in quit rates in the experimental condition versus the control at 6-month follow-up). Finally, we can estimate a 3.7% baseline rate of smoking cessation at 6-month follow-up in a general population sample using findings from the 2008 Canadian Tobacco Use Monitoring Survey (CTUMS) [3]. Given these results, we predict that the subjects in the control condition will display a 3.7% quit rate and subjects in the experimental condition will display a 8.7% quit rate at 6-month follow-up.

Using these assumptions, a power analysis was conducted; this indicated that a total of 403 respondents would be needed per condition to detect a 5% difference at a significance level of .05 with a power of 80% (estimate calculated with continuity correction factored in) [27]. Thus, we have chosen an ideal sample size of approximately 800 respondents in order to conduct appropriate analyses.

Based on our previous research using a similar research design, albeit with problem drinkers rather than smokers [28,29], a 20% attrition between baseline and 6-month follow-up can be expected (the most important follow-up for this study); this would mean that a sample size of 1,000 respondents would be needed at baseline to take part in the randomized trial. Similar follow-up rates have been obtained for smokers in the Ontario Tobacco Survey, indicating that a 20% attrition rate estimate is also reasonable for smokers [30].

From a recent survey of current smokers [10] we know that 62% of 10 or more cigarettes a day smokers say that they would be interested in receiving NRT. Note that the 62% proportion is slightly different from the 58.9% previously reported from this same survey. This is because the proportion reported here for the purposes of generating a sample size estimate was calculated using unweighted data and employs only those smokers from this pilot survey who currently smoke 10 or more cigarettes per day (rather than all smokers who smoked 10 or more cigarettes per day at some point in their life as is employed in the published paper).

Further, from this same survey, we know that 57% of those interested in free NRT say that they would use this NRT to quit, state they would start using it within a week, and would also be willing to have it sent to their homes. Finally, results from this same pilot survey indicate that approximately 14% of those willing to have NRT sent to their homes would screen out due to health contraindications (heart or circulatory problems not including high blood pressure; currently pregnant, intending to become pregnant or breastfeeding) [10]. Note that this estimate of health contraindications is an overly conservative one as the question on heart or circulatory problems asked about lifetime experience. Thus, it is likely that less than 14% of potential subjects will be excluded for this reason. However, we will use the 14% figure in this estimate in order to ensure an adequate sample size.

In conclusion, in order to conduct this trial, we would need to recruit approximately 3,000 respondents at baseline who smoke 10 or more cigarettes per day and who are also willing to take part in an 8-week and a 6-month follow-up with saliva sample collection by mail at each time point (note: saliva samples only collected for the 1,000 participants at baseline who will take part in the randomized trial).

#### Analysis Plan

The primary analyses will employ manual stepwise logistic regressions. Specifically, dependent measures will be 30 day point prevalence abstinence at 6-month follow-up and 7 day point prevalence abstinence at 8-week follow-up. Separate logistic regression analyses will be conducted for each time point (validated by saliva cotinine sample). In the primary analyses, subjects lost to follow-up will be assumed to be active smokers. In Step one of the logistic regression, experimental condition will be entered as a dummy coded variable (0 = control condition; 1 = experimental condition). This step will test the primary hypothesis regarding the impact of sending free NRT on quit rates. Steps two and three of the logistic regression will involve adding interaction terms to test the mediator and moderator hypotheses (3-HC/cotinine ratio, compliance with NRT use, prior use of NRT; main effects terms entered in Step two and interaction terms entered in Step three). Sex difference analyses will be conducted in a similar manner (i.e., is there an interaction effect of subjects' sex by receipt of NRT on abstinence rates at 6-month follow-up?). As part of these primary analyses, we will also conduct a chi-square test to explore whether there is differential loss to follow-up between experimental conditions. Further we will replicate the primary analyses with all subjects with missing data excluded from the analyses. If the results are the same between the analyses where the missing data is excluded and where subjects lost to follow-up are assumed to still be current smokers, then we can safely assume that there is no differential attrition between conditions (or, at least none that impacted on the outcome of the trial).

Subgroup analyses will examine quit rates among subjects within the experimental and control conditions who: (a) are slow versus fast nicotine metabolizers as measured by the 3-HC/cotinine ratio; (b) report using all 5 weeks of the free NRT they were sent as compared with those who do not use all 5 weeks; (c) had used NRT for a quit attempt at least once before participation in the proposed study as compared with those who had never used NRT before; and (d) are male or female (to assess any interaction by sex). Further, we will compare subjects in the experimental condition who used the NRT to just those subjects in the control condition who did not use any NRT during the same time period (i.e., they did not obtain NRT from other sources). Finally, using just the baseline data (N = 1,000), we will relate the 3-HC/cotinine ratio to patterns of smoking and prior use of NRT.

## Discussion

There has been extensive research evaluating the efficacy of NRT as a means to promote smoking cessation. In a systematic review of 132 randomized controlled trials involving NRT, Stead et al. [16] concluded that NRTs increase the rate of quitting smoking by 50 to 70%, irrespective of the clinical setting in which the smoker is treated. The Public Health Service in the U.S. has, over the years, examined over 8,700 research articles in order to prepare guidelines for the treatment of tobacco use and dependence [26]. They found that medications such as NRTs are effective in increasing long-term smoking abstinence rates and recommend that clinicians encourage their use. They also found evidence that health care policies, for example, insurance coverage of smoking cessation treatments, play an important role in the likelihood that smokers will receive effective smoking cessation treatment and thus successfully quit.

With the evidence supporting the efficacy of NRTs in successful smoking cessation and with the recommendations from these reviews promoting increased access to NRT for smokers, the undertaking of mass distribution of free NRT appears to be well founded. However, there have been no randomized controlled trials yet of mass distribution of free NRTs to show whether or not such an initiative would indeed result in a population level quit rate above that attained without this intervention. The present study would be the first such trial. In particular, employing a panel survey design, this randomized controlled trial will evaluate the effectiveness of free mass distribution of the nicotine patch to Canadian smokers interested in free NRT. In contrast to subjects in the experimental condition receiving a 5-week program of nicotine patches, subjects in the control condition will not be offered free nicotine patches. At 8-week and 6-month follow-ups, comparison of smoking outcomes between the two groups will evaluate the effectiveness of free NRT intervention and a naturalistic progression of smokers interested in quitting but not receiving such intervention.

The proposed trial will be useful to policy makers, in particular Canadian policy makers, in the development of public health initiatives to reduce the prevalence of smoking. If the project finds support for the effectiveness of supplying interested smokers with free NRT in order to help them quit, it would provide evidence to move forward with policies designed to make NRT treatment readily and freely available to smokers who request it.

## Competing interests

The authors declare that they have no competing interests. Dr. Tyndale owns shares and participates in Nicogen Research Inc., a company focused on novel smoking cessation treatment approaches. No Nicogen funds were used in this work and no other Nicogen participants reviewed the manuscript. Dr. Tyndale has also consulted for Novartis.

## Authors' contributions

All authors have made an intellectual contribution to this research trial. JAC is the principal investigator of the trial, with overall responsibility for the project. He conceived the study, prepared the protocol, and oversees all phases of the project. STL specializes in advanced multivariate statistical modeling, and as such, he is the biostatistician on this project. He has also provided insight pertaining to the potential mechanism for disseminating the research results to decision-makers given his professional roles within different government organizations (e.g., Cancer Care Ontario, Canadian Institutes of Health Research) and stakeholder groups (e.g., Canadian Cancer Society). PLS and LZ have provided expertise in mass distribution of NRT. They have provided leadership with respect to the design of the study as well as to the analytical approaches to be employed. RFT oversees the collection and analysis of saliva samples collected at baseline, 8-week, and 6-month follow-up. She has provided leadership on the analysis and interpretation of the 3-HC/cotinine ratio moderator hypothesis as well as the validation of self-report by saliva cotinine levels. VK is the trial manager and is responsible for coordination of the trial. All authors have contributed to the drafting process, and all authors have read and approved the final manuscript.

## Pre-publication history

The pre-publication history for this paper can be accessed here:

http://www.biomedcentral.com/1471-2458/11/741/prepub
